# The relationship between mitochondrial respiration, resting metabolic rate and blood cell count in great tits

**DOI:** 10.1242/bio.060302

**Published:** 2024-03-11

**Authors:** Elisa Thoral, Carmen C. García-Díaz, Elin Persson, Imen Chamkha, Eskil Elmér, Suvi Ruuskanen, Andreas Nord

**Affiliations:** ^1^ Lund University, Department of Biology, Section for Evolutionary Ecology, Sölvegatan 37, SE-223 62 Lund, Sweden; ^2^ Lund University, Department of Clinical Sciences, Mitochondrial Medicine, Sölvegatan 17, SE-221 84, Lund, Sweden; ^3^ Department of Biological and Environmental Science, University of Jyväskylä, FI-40014 Jyväskylä, Finland

**Keywords:** Erythrocyte, Oxidative metabolism, Resting metabolic rate, Basal metabolic rate, Great tit, Mitochondria

## Abstract

Although mitochondrial respiration is believed to explain a substantial part of the variation in resting metabolic rate (RMR), few studies have empirically studied the relationship between organismal and cellular metabolism. We therefore investigated the relationship between RMR and mitochondrial respiration of permeabilized blood cells in wild great tits (*Parus major* L.). We also studied the correlation between mitochondrial respiration traits and blood cell count, as normalizing mitochondrial respiration by the cell count is a method commonly used to study blood metabolism. In contrast to previous studies, our results show that there was no relationship between RMR and mitochondrial respiration in intact blood cells (i.e. with the ROUTINE respiration). However, when cells were permeabilized and interrelation re-assessed under saturating substrate availability, we found that RMR was positively related to phosphorylating respiration rates through complexes I and II (i.e. OXPHOS respiration) and to the mitochondrial efficiency to produce energy (i.e. net phosphorylation efficiency), though variation explained by the models was low (i.e. linear model: *R*^2^=0.14 to 0.21). However, unlike studies in mammals, LEAK respiration without [i.e. L(n)] and with [i.e. L(Omy)] adenylates was not significantly related to RMR. These results suggest that phosphorylating respiration in blood cells can potentially be used to predict RMR in wild birds, but that this relationship may have to be addressed in standardized conditions (permeabilized cells) and that the prediction risks being imprecise. We also showed that, in our conditions, there was no relationship between any mitochondrial respiration trait and blood cell count. Hence, we caution against normalising respiration rates using this parameter as is sometimes done. Future work should address the functional explanations for the observed relationships, and determine why these appear labile across space, time, taxon, and physiological state.

## Teaser

**Summary:** Our results show that the relationship between resting metabolic rate and blood mitochondrial metabolism depends on the level of cellular permeabilization as well as the level of activation of the electron transport chain. No relationship was detected between blood mitochondrial respiration and blood cell count in our conditions.

## INTRODUCTION

Energy spent by individuals to fuel physiological and behavioral processes can be estimated by measuring metabolic rate (Norin and Metcalfe, 2019). In endotherms, the minimum energy consumption needed at rest in a post-absorptive state is referred to as resting metabolic rate (RMR), or basal metabolic (BMR) if the measurements are performed in thermoneutrality (Bligh and Johnson, 2001). For the purposes of this article, we will always refer to the metabolic rate measured as RMR. RMR varies across environmental, temporal, and spatial contexts. For example, tropical birds are known to have a lower RMR compared to temperate species (Wiersma et al., 2007). On smaller geographic scales, RMR varies predictably with environmental conditions such as temperature and photoperiod (McKechnie and Swanson, 2010; Swanson and Vézina, 2015; White et al., 2007).

Recent research has identified a large need to understand how and why metabolic rate varies among and within individuals (Metcalfe et al., 2023). One candidate explanation is changes in mitochondrial metabolism. At the cellular level, the vast majority of energy that is subsequently used to fuel RMR is produced by oxidative phosphorylation (OXPHOS) in the mitochondria (Lehninger et al., 1993). Mitochondria use oxygen and energy substrates to transport electrons from complex I to IV (cytochrome c oxidase) of the electron transport system (ETS) while simultaneously forming a proton gradient across the inner mitochondrial membrane. Chemical energy in the form of adenosine triphosphate (ATP) is formed when this proton motive force is dissipated through complex V (ATP synthase) at the end of the ETS (Lanza and Nair, 2009). However, the coupling between oxygen consumption and ATP production is not complete, such that a part of mitochondrial respiration is used to counteract proton leakage back into the mitochondrial matrix (i.e. LEAK respiration). This causes energy contained in the proton gradient to be released as heat, which is the basis for non-shivering thermogenesis in the brown adipose tissue of mammals (e.g. Jastroch et al., 2010). The ETS also produces reactive oxygen species, which can result in oxidative stress if not quenched by the body's antioxidant defences (Munro and Treberg, 2017, but see Chung and Schulte, 2020).

Because metabolic rate at the whole-animal and cellular levels seem to respond similarly to environmental perturbations (Milbergue et al., 2022; Sokolova, 2018 but also see Thoral et al., 2022, 2023), mitochondrial metabolism could be a good candidate pathway to explain the mechanistic underpinnings of variation in organismal metabolism (Koch et al., 2021; Swanson et al., 2014; White and Kearney, 2013). In line with this, RMR is positively related to the LEAK respiration and the activity of cytochrome c oxidase in several tissues (including muscle, liver and kidney) in the Chinese hwamei (*Garrulax canorus*) (Wang et al., 2019), and LEAK respiration may explain some 20-25% of thermoneutral RMR in mammals under constant conditions (Porter and Brand, 1995; Rolfe and Brand, 1996). Moreover, in European starlings (*Sturnus vulgaris*), RMR is positively related to respiration on endogenous substrates in intact red blood cells (i.e. the ROUTINE respiration) and this relationship was stronger than that between RMR and OXPHOS respiration in permeabilized cells in a range of other tissues (Casagrande et al., 2023). In great tits (*Parus major*), RMR is also positively related to ROUTINE respiration, at least when the birds are not stressed (i.e. have low circulating levels of corticosterone; Malkoc et al., 2021). The relationship between mitochondrial respiration and organismal metabolic rate may also vary depending on which parts of the ETS are addressed. For example, in black-capped chickadees (*Poecile atricapillus*), RMR in thermoneutrality is positively correlated with OXPHOS respiration in the liver, but not with the LEAK respiration (Milbergue et al., 2022). Thus, the level of activation of the ETS can influence how cellular metabolism is related to organismal metabolic rate.

The observed relationship between RMR and mitochondrial metabolism in blood cells (Casagrande et al., 2023; Malkoc et al., 2021) is encouraging, because mitochondrial function in blood can be assessed using minute samples that can be safely collected from small organisms and under field conditions (Nord et al., 2023; Stier et al., 2019). Therefore, blood is a good candidate tissue for studying the relationship between mitochondrial metabolism and individual metabolic rate, and how this relationship could be affected by the physiological conditions in which the measurements are made. Here, we used data collected on wild great tits (*Parus major* L.) to explore how the relationship between RMR and mitochondrial respiration traits varies between respiratory states. Specifically, the mitochondrial measurements were performed using cells respiring only on endogenous (intracellular) substrates (i.e. ROUTINE) which were subsequently permeabilized in the presence of saturating concentrations of mitochondrial substrates and adenosine diphosphate (ADP). This allowed us to explore the link between organismal and cellular metabolism both in presumably representative *in vivo* conditions (ROUTINE respiration in intact cells) and in standardized conditions when substrate availability did not constrain respiration (after the permeabilization). Our purposes were twofold: (1) to investigate to what extent blood cell respiration was indicative of organismal RMR, for which there is currently mixed evidence, and (2) to explore whether the contribution of blood mitochondrial respiration towards variation in RMR changed depending on physiological contexts. In line with previous findings (Casagrande et al., 2023; Milbergue et al., 2022), we predicted that both respiration in intact cells (i.e. ROUTINE). and phosphorylating respiration in permeabilized cells (i.e. OXPHOS) should correlate with RMR. We also predicted that LEAK respiration should explain part of the variation in RMR, as observed in mammals (see above). An ancillary goal was also to investigate the relationship between blood mitochondrial respiration and blood cell count. Because the relationship between cell count and blood cell respiration is expected, normalization of mitochondrial respiration in blood cells by cell count is sometimes used (e.g. Cossin-Sevrin et al., 2023; Stier et al., 2019), even though there are no published data underlying this association.

## RESULTS

### Relationship between mitochondrial respiration and cell count

**Fig. 1.**
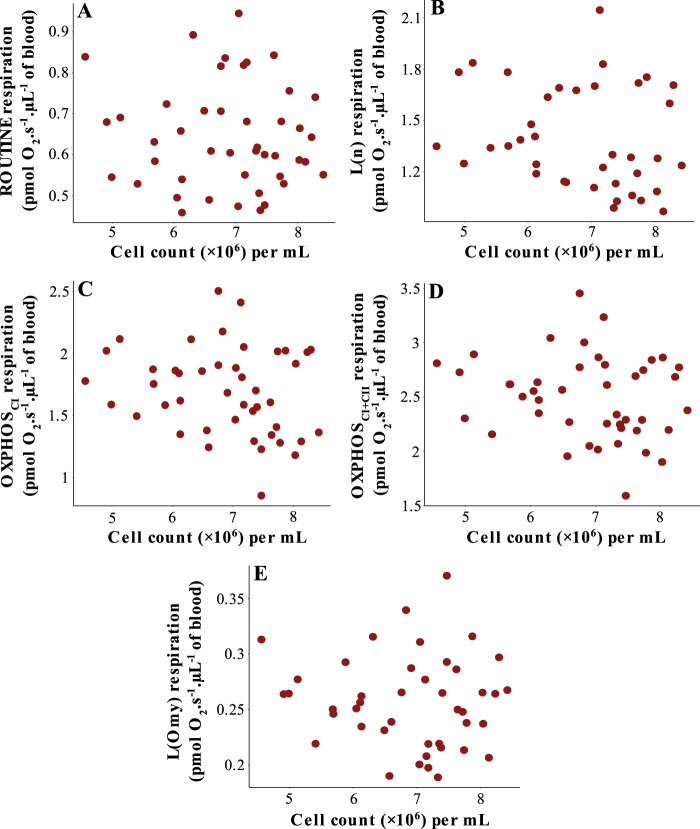
**Mitochondrial respiration rates as a function of total cell count in great tits blood cells.** (A) ROUTINE respiration, that is, mitochondrial respiration on endogenous intracellular substrates; (B) L(n) (i.e., non-phosphorylating) respiration after the cell membrane was permeabilized by digitonin, but before the addition of substrates for mitochondrial complexes I and II; (C) OXPHOS_CI_ respiration, that is, phosphorylating respiration when complex I was activated by malate and pyruvate; (D), OXPHOS_CI+II_ respiration, that is, phosphorylating respiration through complexes I and II in the presence of malate, pyruvate and succinate; (E) L(Omy) respiration, that is, non-phosphorylating respiration after complex V (ATP synthase) was inhibted by oligomycin. Apart from ROUTINE, all respiration states were assessed in permeabilized cells in the presence of saturating amounts of substrates or inhibitors as detailed in Table 1.

Total cell count obtained using the TC-20 varied 1.8-fold (from 4.56×10^6^ to 8.42×10^6^ cells/ml) between individuals for the same blood volume. Yet, there were no relationships between any mitochondrial respiration traits and cell count when outliers were excluded from the analyses (Fig. 1, Table 2). Nor was there any relationship between non-mitochondrial respiration rate (i.e. ROX respiration) and blood cell count (Fig. S2).

**Table 1.**
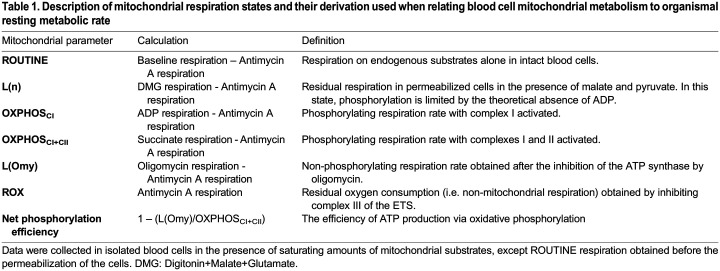
Description of mitochondrial respiration states and their derivation used when relating blood cell mitochondrial metabolism to organismal resting metabolic rate

**Table 2.**
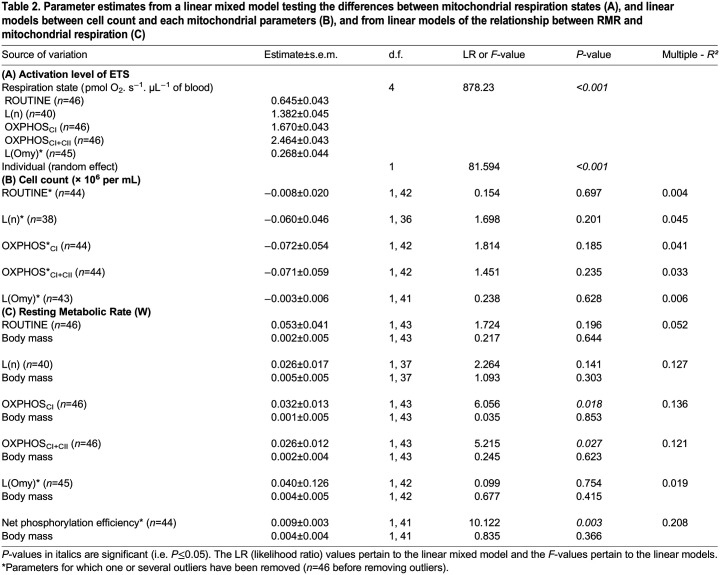
Parameter estimates from a linear mixed model testing the differences between mitochondrial respiration states (A), and linear models between cell count and each mitochondrial parameters (B), and from linear models of the relationship between RMR and mitochondrial respiration (C)

### Relationship between RMR and mitochondrial respiration

We only present thermoneutral RMR data in the main paper (i.e. RMR_20°C_), because RMR in the two temperatures were strongly correlated (Pearson correlation coefficient R=0.56; Fig. S3). Accordingly, the slopes of the relationships between RMR and mitochondrial respiration traits never differed significantly between the two night temperatures (respiration trait×measurement temperature: all *P*>0.3). Figures and statistics pertaining to the RMR_8°C_ data are reported in the ESM (Fig. S3; Table S1).

**Fig. 2.**
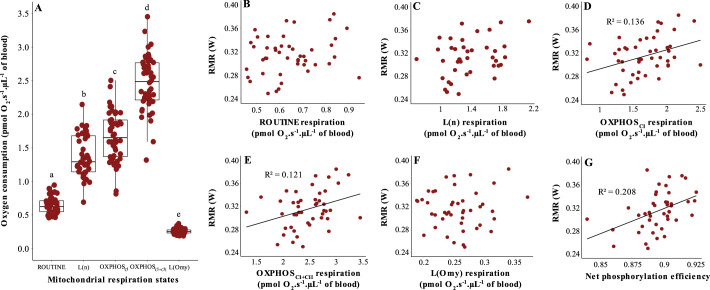
**Mitochondrial respiration in great tit blood cells and its relationship to resting metabolic rate.** (A) Oxygen consumption of mitochondria from great tit blood cell in different respiration states before (i.e. ROUTINE respiration) and after cell permeabilization. (B-G) The relationship between resting metabolic rate (RMR) and mitochondrial respiration traits measured in blood cells. Apart from ROUTINE respiration (B), all respiration states were assessed in permeabilized cells in the presence of saturating amounts of substrates or inhibitors as detailed in Table 1. Solid lines show significant relationships (*P*≤0.05) whereas the absence of a regression line indicates non-significant relationships. Different letters in A show significant differences.

Mitochondrial oxygen consumption was significantly different in all the respiration states we measured (*P*<0.001, Fig. 2A, Table 2). There was no relationship between RMR and ROUTINE, that is, in intact cells where the mitochondria respire on endogenous substrates (*P*=0.196; Fig. 2B). However, after permeabilizing the cells, we found that variation in RMR at 20°C (i.e. at thermoneutrality) was significantly explained by several mitochondrial parameters. Specifically, there were significant positive relationships between RMR and OXPHOS respirations through both complex I and complexes I+II (OXPHOS_CI_: *P*=0.018, OXPHOS_CI+CII_: *P*=0.027, Fig 2D,E, Table 2) as well as between RMR and net phosphorylation efficiency (*P*=0.003; Fig. 2G, Table 2). However, neither L(n) nor L(Omy) were related to RMR at 20°C [L(n): *P*=0.141; L(Omy): *P*=0.754, Table 2, Fig. 2C,F, respectively].

The relationships between mitochondrial parameters and RMR_8°C_ were similar to those for RMR_20°C_, except for the net phosphorylation efficiency, which was no longer significant (*P*=0.193; Fig. S4F, Table S1).

### The effects of outliers

Results with outliers included are presented in the Supplementary Material (Figs S5 and S6, Table S2). In the cell count analyses, L(n), OXPHOS_CI_ and OXPHOS_CI+II_ respirations decreased significantly with cell count when two outliers (with a count>17.1×10^6^ cells/ml) where retained in the analyses (0.005≥*P*≤0.021, Fig. S6). Keeping outliers did not change relationships between RMR and mitochondrial respiration, except for the net phosphorylation efficiency that was no longer significantly related to RMR_20°C_ (*P*=0.160, Fig. S5).

## DISCUSSION

RMR was not significantly related to mitochondrial respiration before the cells were permeabilized (i.e. to the ROUTINE respiration), contrary to what has been found in a previous bird study (Casagrande et al., 2023; see also Malkoc et al., 2021). However, once the cells had been permeabilized, we found positive relationships between RMR and mitochondrial respiration, but these depended on which complexes of the ETS were activated.

OXPHOS respiration through both complex I and complexes I+II was positively related to RMR at both temperatures (Fig. 2D,E; Fig. S4C,D). Similar results were observed by Milbergue et al., (2022), where OXPHOS_CI_ measured in liver cells was positively correlated to RMR measured at −10°C (but unrelated to RMR measured at 27°C). The relationship between RMR and OXPHOS respiration could simply reflect that more ATP must be produced by the mitochondria in an animal that is metabolizing more energy (Koch et al., 2021) and that, in the case of our great tits, seemed to be achieved by consuming more oxygen rather and, at least in thermoneutrality, by making the mitochondria more effective, as indicated by the positive relationship between RMR and net phosphorylating respiration (Fig. 2G). Another, not mutually exclusive, possibility is that ATP produced in the blood is involved in the maintenance of basal functions of tissues, for example, by stimulating vasodilatation and improving the dissociation of oxygen from haemoglobin in target tissues (Ellsworth and Sprague, 2012; Misiti et al., 2022). The many possible roles of ATP produced by blood is still poorly understood in birds and we believe this to be an exciting avenue for future investigation.

There was no relationship between RMR and any LEAK respiration, neither with [L(Omy)] or without [L(n)] adenylates (Fig. 2C,F). This contrasts mammal studies where L(Omy) in hepatocytes accounted for some 20-25% of the variation in RMR in post-absorptive mice (Brand et al., 1999; Rolfe et al., 1999). It is possible that the explanation for the difference between studies is that the contribution of L(Omy) to RMR depend on the tissue studied. For example, Salin et al., (2016) found that standard metabolic rate [SMR; i.e. minimum MR during minimum activity in resting ectotherms (Bligh and Johnson, 2001)] in brown trout (*Salmo trutta*) was correlated to L(Omy) in liver cells, but not in skeletal muscle where L(Omy) was instead correlated to maximal exercise-induced MR. Similarly, in cold-acclimated birds, muscle L(Omy) was correlated to maximum cold-induced metabolic rate (Milbergue et al., 2022). Moreover, a study made on isolated mitochondria from human skeletal muscle also found no relationship between RMR and L(n), and instead suggested that RMR is determined more by mitochondrial density and oxygen affinity (Larsen et al., 2011).

Using a previously published method to count the total number of cells in bird blood samples for mitochondrial studies, we found no relationships between cell count and any respiration trait. We have obtained similar results in studies of Japanese quail (*Coturnix japonica*, Thoral, E. et al., in prep). We expect such a relationship to be present if different blood volumes from the same individual are added to a preparation, but we show here that it does not present between birds, despite 1.8-fold variation in cell count across subjects in the study. While it is conceivable that an increase in the number of cells should correlate with mitochondrial content in a tissue, cell count alone is not necessarily the sole, or strongest, determinant thereof. Rather, we foresee strong contributions to volume-adjusted respiration from, for example, the proportion of red blood cells compared to other blood cells, cell volume, and quality and quantity of the mitochondria contained in these cells. On this basis, we caution against using blood cell count to normalize mitochondrial respiration without prior verification against known markers of mitochondrial content, however intuitive this relationship may be. Several other metrics are commonly used to for normalizing mitochondrial respiration to the quantity of mitochondria, such as citrate synthase activity (Milbergue et al., 2022; Dawson and Salmón, 2020; Nord et al., 2021), cytochrome c oxidase activity (Milbergue et al., 2022; Salin et al., 2018), and mitochondrial DNA copy number (Stier et al., 2019). Studies should be carried out to determine which of these, and other putative (see Larsen et al., 2012), markers is the most appropriate for avian blood cell mitochondria.

This study revealed that there are relationships between RMR and some mitochondrial respiration traits in blood cells, but that these need not be apparent when assaying intact cells respiring on (limited) endogenous substrates (i.e. ROUTINE). This contrasts other recent studies were RMR and ROUTINE were strongly correlated (0.70; Casagrande et al., 2023). However, by permeabilizing cells to address mitochondrial function at saturating substrate availability, we found that RMR was positively correlated to phosphorylating respiration and effectiveness. We do not know if these relationships were causal or correlated. The latter might be more likely since blood cells are less aerobic, and contribute less to RMR, compared to more metabolically active tissues such as liver and muscle (Casagrande et al., 2023). Indeed, the variation in RMR explained by phosphorylating respiration states in our study (i.e. *R*^2^=0.14–0.21) was notably lower than reported for OXPHOS_CI_ in liver cells in a similar bird model (0.39; Milbergue et al., 2022). This suggests that caution must be exercised when inferring RMR from measurement of mitochondrial respiration in single tissues, which is deceivingly simple but likely provides imprecise and oversimplified estimates of metabolic rate at best (see Thoral et al., 2024). Yet, mitochondrial metabolism in blood cells correlates with that in other tissues such as the brain, skeletal muscle, and liver (Casagrande et al., 2023; Stier et al., 2017, 2022; Thoral, E. et al., in prep). It also shows predictable responses to both environmental or physiological stressors (García-Díaz et al., 2023; Nord et al., 2021) and life history parameters such as reproductive effort (Stier et al., 2019) and aging (Dawson and Salmón, 2020; Salmón et al., 2023). These studies reiterate the utility of blood cell measurement in ecology and evolution, but serve as a reminder of the fact that we still know very little about the role of mitochondria in the erythrocytes in non-mammal vertebrates. Future studies are now needed to shed light on why there is a relationship between blood cell respiration and RMR, when blood cell measurements are a useful approximation of organismal biology, and why the strength and nature of these relationships seem to vary within and between species, over time and space.

## MATERIALS AND METHODS

Great tits (*n*=62; 24 females, 37 males, 1 unknown) were captured from December 2021 to March 2022 when roosting in nest boxes at night starting at least 1 h after sunset in three sites within close proximity and with similar habitat characteristics in southernmost Sweden: Linnebjer (55° 43′N 13° 17′E; *n*=7), Frueräften (55° 72′N, 13° 31′E; *n*=8) and Vomb (55° 39′N, 13° 33′E; *n*=59). The birds were returned to the nest box in which they had been caught at, or just after, sunrise the next day.

### Resting metabolic rate

The birds were brought to Lund University within 3 h of capture. The birds were put into a 1 L hermetically sealed glass container ventilated with dry (drierite) air at 400–500 ml/min (standard temperature and pressure, dry, STPD; registered using a FB-8 mass flow meter from Sable Systems, Las Vegas, NV, USA) and contained inside a climate chamber in complete darkness (Weiss Umwelttechnik C180, Reiskirchen, Germany). This flow rate was sufficient to keep CO_2_ in outgoing air below 0.5% (measured using a CA-10 carbon dioxide analyzer; Sable Systems). Temperature inside the respirometry chambers was recorded using a TC-2000 thermocouple box (Sable Systems) fitted with fine-wire (36 G) type T (copper-constantan) thermocouples with the temperature-sensitive junction contained in the chamber roof where they were minimally affected by heat production by the bird. The resting metabolic rate (RMR) was then measured firstly in thermoneutrality (20.06±0.17°C) for 4 h and then below thermoneutrality at 8°C (7.68±0.19°C) for 4 h. The fraction of oxygen in outgoing air was measured in dry, CO_2_-free (drierite, ascarite) air using a FC-10 oxygen analyzer (Sable Systems). The temperature decrease was 6.25°C per h during the transition between the warm and mild temperature. We sub-sampled 150 ml/min STPD from the main gas stream (which was 400–500 ml/min STPD) using a SS4 sub-sampler (Sable Systems). O_2_ was measured in dry, but not CO_2_-free air, and so we mathematically accounted for the effect of CO_2_ on O_2_ when calculating metabolic rate (Lighton, 2019). Four birds were measured sequentially for 10 min each throughout the night. Each such measurement cycle started and ended with a 10 min recording of reference (‘baseline’) air. We excluded the first two measurement cycles from further analyses to reduce any effects of handling stress on our estimated RMR. The rate of oxygen consumption (*V̇*O_2_) was then calculated based on the most stable 2 min of each 10 min recording in the remaining data, using Eqn 11.7 in Lighton (2019) and was subsequently converted to metabolic rate assuming an energy equivalence of 20 J per ml O_2_ (Kleiber, 1961). We defined the single lowest *V̇*O_2_ for an individual in each of the temperatures used as RMR (i.e. each bird was assigned a RMR_20°C_ and a RMR_8°C_).

### Mitochondrial measurement

The birds were bled (≤10% of blood volume) from the jugular vein within 1 min of removal from the metabolic chambers in the morning after RMR measurements, and the sample was stored in 4 ml Na-heparin tubes (BD Vacutainer, Franklin Lakes, NJ, USA) at 10–12°C until processed 30 min to 2.5 h later. Our previous work shows that mitochondrial respiration (in intact cells) remains largely unaltered over 24 h when samples are stored cold (Nord et al., 2023). Mitochondrial measurements were performed in permeabilized cells, at a representative bird body temperature (41°C, Prinzinger et al., 1991) using high-resolution respirometers (Oxygraph O2k high-resolution respirometers, Oroboros Instruments, Innsbruck, Austria) following Nord et al., 2023. First, 40 µl of blood were added to 1 ml of ice-cold respiration medium (MiR05: 0.5 mM EGTA, 3 mM MgCl_2_, 60 mM K-lactobionate, 20 mM taurine, 10 mM KH_2_PO_4_, 20 mM HEPES, 110 mM sucrose, 1 g/L free-fatty-acid bovine serum albumin, pH 7.1) and mixed gently. 50 µl of this mixture was transferred to a new tube containing 950 µl of PBS, which was stored at 5°C for 10 to 30 min, after which we vortexed the sample and counted total cell number using a TC20 Automated Cell Counter (Bio-Rad, Solna, Sweden) in attempt to normalize mitochondrial respiration for cell count using the same methodology as in other studies of bird blood mitochondria (Cossin-Sevrin et al., 2023; Stier et al., 2019).

The remaining blood-MiR05 tube was centrifuged for 2 min at 1000 RCF and the supernatant was removed. The pellet was then resuspended in 1 ml of 41°C MiR05 from the respirometry chamber and the entire solution was put back into the chamber to reach a final volume of 2.1 ml. Once ROUTINE (i.e. respiration on endogenous substrates before permeabilization, see Table 1) had been measured, digitonin (chamber concentration: 20 µg/ml) was added to permeabilize the cells. Once oxygen consumption had stabilized after digitonin addition, we added malate (5 mM) and pyruvate (2 mM) to fuel complex I of the ETS and measure a basal oxygen consumption [i.e. LEAK state without adenylates or L(n), see Table 1]. Then, we stimulated the production of ATP through complex I (i.e. OXPHOS_CI_, see Table 1) by adding ADP (1.25 mM). When the response had stabilized, we stimulated complex II by adding 10 mM succinate to obtain maximal phosphorylating respiration rate (i.e. OXPHOS_CI+II_, see Table 1). Then, oligomycin (2.5 µM) was added to inhibit ATP synthase. Since ADP cannot be phosphorylated to ATP in the presence of oligomycin, the remaining oxygen consumption is mostly used to counteract for proton leakage across the inner mitochondrial membrane [i.e. LEAK state with oligomycin or L(Omy), see Table 1]. Finally, antimycin A (5 µM) was added to inhibit complex III, after which any remaining respiration is of non-mitochondrial original (i.e. residual oxygen consumption, or ROX respiration, see Table 1). ROX was removed from all other respiration rates before analyses. Raw data from a representative experiment is presented in the Fig. S1.

### Data handling and statistical analyses

We calculated the net phosphorylation efficiency, which is an index of the mitochondrial efficiency to produce ATP by oxidative phosphorylation (Shama et al., 2016) to relate metabolic rate to functional aspects of mitochondrial respiration. The calculation method and definitions of the different mitochondrial parameters are detailed in Table 1.

At the individual level, eight measures of RMR at 8°C are missing due to technical issues. We also removed two RMR measurements obtained at thermoneutrality since the gas consumption curves clearly deviated from the normally stable state in a resting individual, instead being notably uneven which is indicative of activity. Thus, these birds did not conform to the resting criterion of RMR (Bligh and Johnson, 2001; RMR>population mean +2 standard deviations). Four other birds were removed from all analyses due to technical issues that caused the experiment to end prematurely and prevent the calculation of RMR_20°C_. At the mitochondrial level, we excluded samples where either oligomycin or ADP did not affect mitochondrial respiration, when overall respiration was close to 0 or when the signal was not stable at all during the measurement (*n*=10 of 62). Data on L(n) respiration are missing for six individuals since ADP was added directly after pyruvate and malate, preventing the measurement of L(n) respiration alone.

All statistical tests were done using R v.4.2.1 (R Development Core Team 2022). We analyzed the effect of the activation level of the ETS using linear mixed model lmer() function in lme4 package (Bates et al., 2015), with the activation level of the ETS as the dependent variable and individuals as a random effect. Tests of the fixed effect was performed using the Anova() function in the car package, and the significance of the random effect was assessed using ranova() from the lmerTest package (Kuznetsova et al., 2017). Likelihood ratios were obtained using the drop1() function from the stats package. Pairwise *post-hoc* comparisons were performed using the pairs() function from the emmeans package (Lenth, 2018). Linear models (lm in R base) were fitted to estimate the effects of mitochondrial respiration parameters [ROUTINE, L(n), OXPHOS and L(Omy) respirations and net phosphorylation efficiency] on RMR. We included body mass as a covariate in all models since it is often an important predictor of metabolic rate. We used linear models (lm in the stats package) to analyze the relationship between cell count and mitochondrial respiration.

All data met the assumptions of normality and homogeneity of variance as determined by examination of diagnostic plots of the final models. The level of significance was set at *P*≤0.05. All analyses were performed with and without outliers (i.e. observations that deviated ≥2 standard deviations from the group mean).

## Supplementary Material

10.1242/biolopen.060302_sup1 Supplementary information
