# Supplementary material for: The relationship between mitochondrial respiration, resting metabolic rate and blood cell count in great tits
Source: Biol Open. 2024 Mar 11;13(3):bio060302. doi: 10.1242/bio.060302 (PMC10958200; doi:10.1242/bio.060302)
Supplement: Supplementary information [file biolopen-13-060302-s1.pdf]

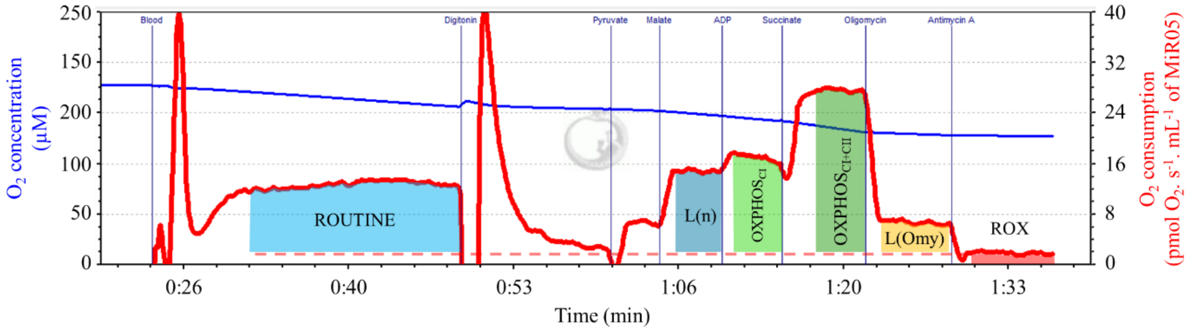

Fig. S1. A representative experiment with permeabilized great tit blood cells. The blue curve and lefthand axis show oxygen concentration in the respirometer chamber (µM of O_2). The red curve and righthand axis show the oxygen consumption (pmol of O_2. s^{-1}. mL^{-1}) of the blood and respiration medium (MiR05). A whole blood sample (40 µL) was centrifuged in 1 mL MiR05 for 2 min at 1000 RCF and the supernatant (containing the plasma) was removed. The pellet was then resuspended in 1mL of 41°C MiR05 from the respirometry chamber and the entire solution was put back into the chamber to obtain a final volume of 2.1 mL ("Blood" in the figure). Once ROUTINE (i.e., mitochondrial respiration on endogenous substrates) had been measured, digitonin (chamber concentration: 20µg/mL; "Digitonin") was added to permeabilize the cells. Once oxygen consumption had stabilised after digitonin addition, we added pyruvate (2mM; "Pyruvate") and malate (5mM; "Malate") to fuel complex I of the ETS to obtain a basal oxygen consumption (*i.e.*, L(n) respiration). Then, we stimulated the production of ATP through complex I (*i.e.*, $OXPHOS_{CI}$) by adding ADP (1.25mM; "ADP"). When the response had stabilised, we stimulated complex II by adding 10 mM of succinate ("Succinate") to obtain maximal phosphorylating respiration rate (*i.e.*, $OXPHOS_{CI+II}$). L(Omy) respiration was measured by injection of oligomycin (2.5µM; "Oligomycin"), after which we inhibited complex III by addition of antimycin A (2.5µM; "Antimycin A"). Any remaining respiration after Antimycin A addition is of non-mitochondrial original (*i.e.*, residual oxygen consumption, or ROX). ROX was removed from all respiration rates before analyses.

Biology Open • Supplementary information

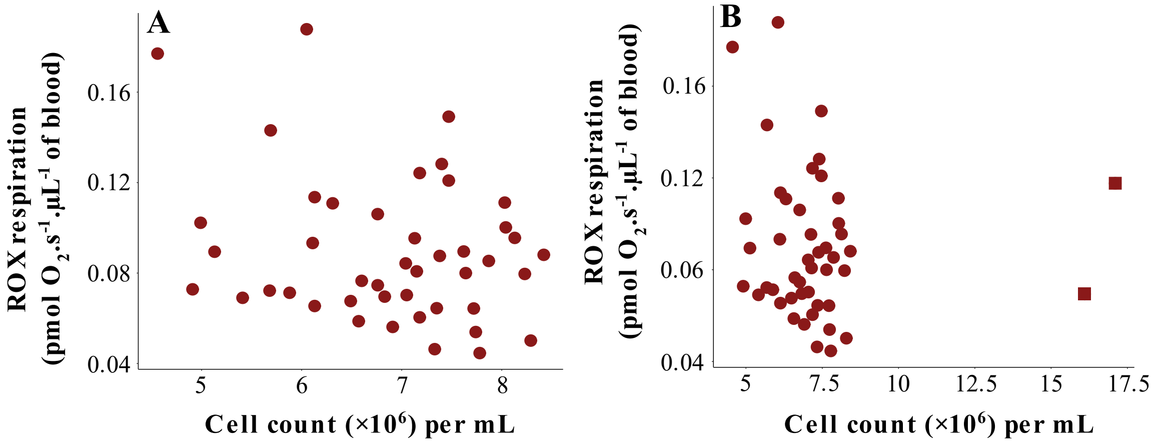

Fig. S2. Non-mitochondrial respiration (i.e., ROX respiration obtained after the injection of antimycin A) as a function of cell count without (A) and with (B) outliers. An observation was considered to be an outlier if it was more than 2 standard deviations from the group mean and are plotted as squares.

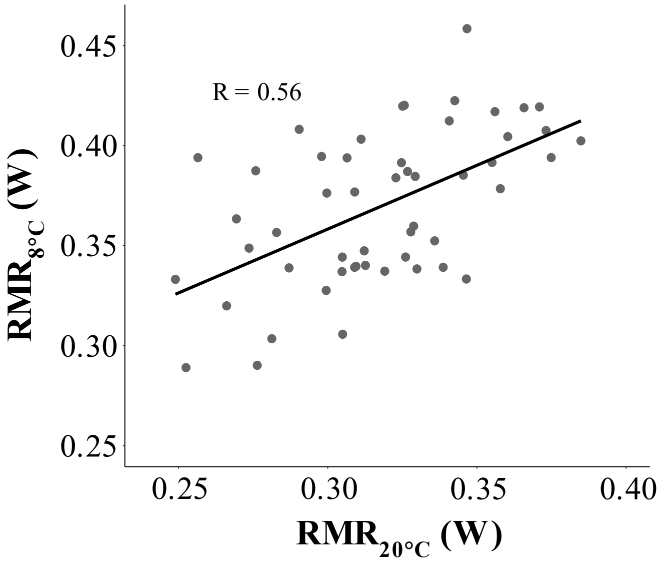

Fig. S3. Resting metabolic rate at 8°C ($RMR_{8°C}$) was significantly related to $RMR_{20°C}$ (Pearson correlation test: $t = 4.74$, $df = 50$, $P < 0.001$, $R = 0.56$).

Biology Open • Supplementary information

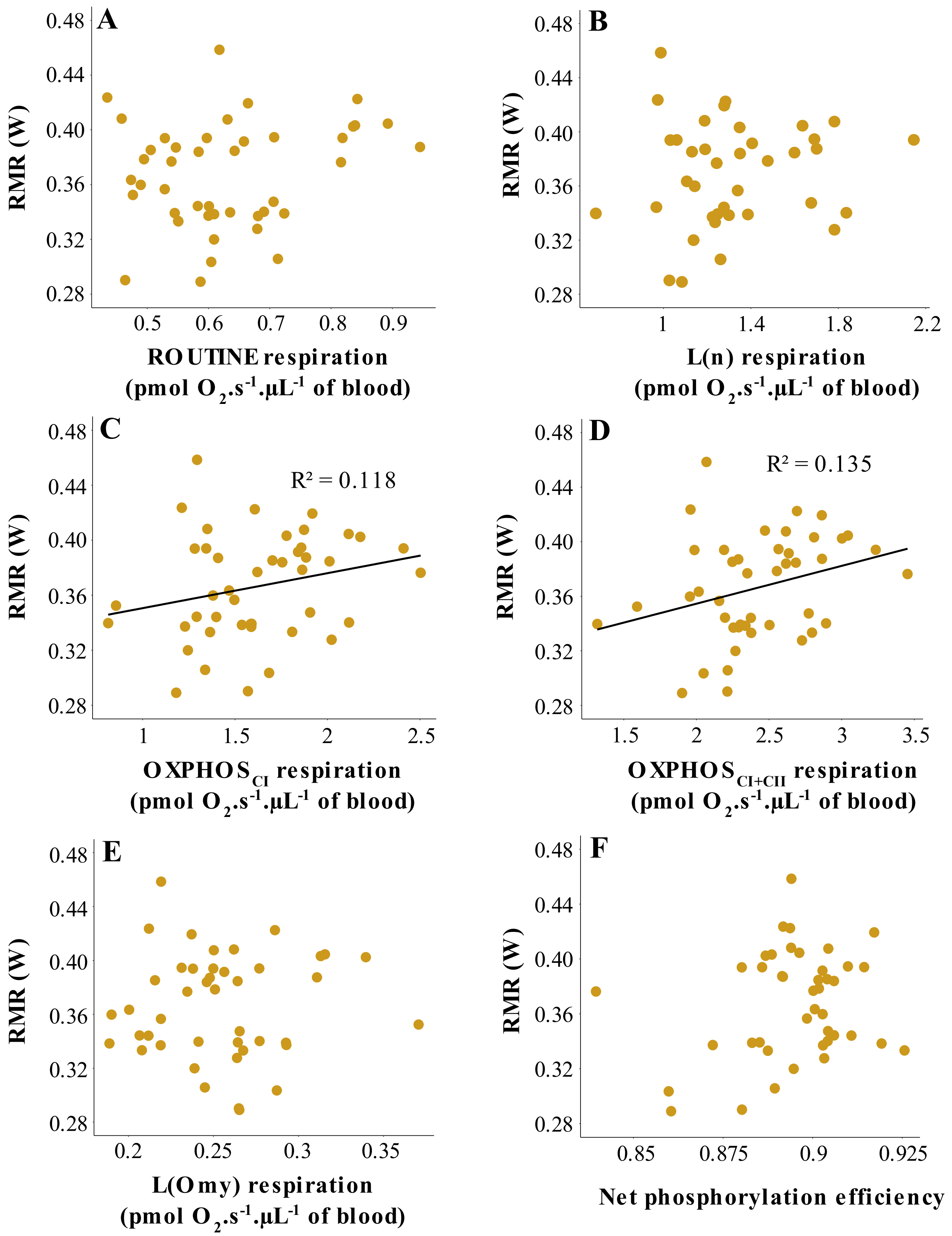

Fig. S4. Relationship between the resting metabolic rate (RMR) at 8°C and mitochondrial respiration parameters obtained in permeabilized blood cells the morning after RMR measurement in great tits. Results without outliers (i.e., observations < 2 standard deviations from the group mean) are presented. Solid lines show significant relationships ($P \leq 0.05$).

Biology Open • Supplementary information

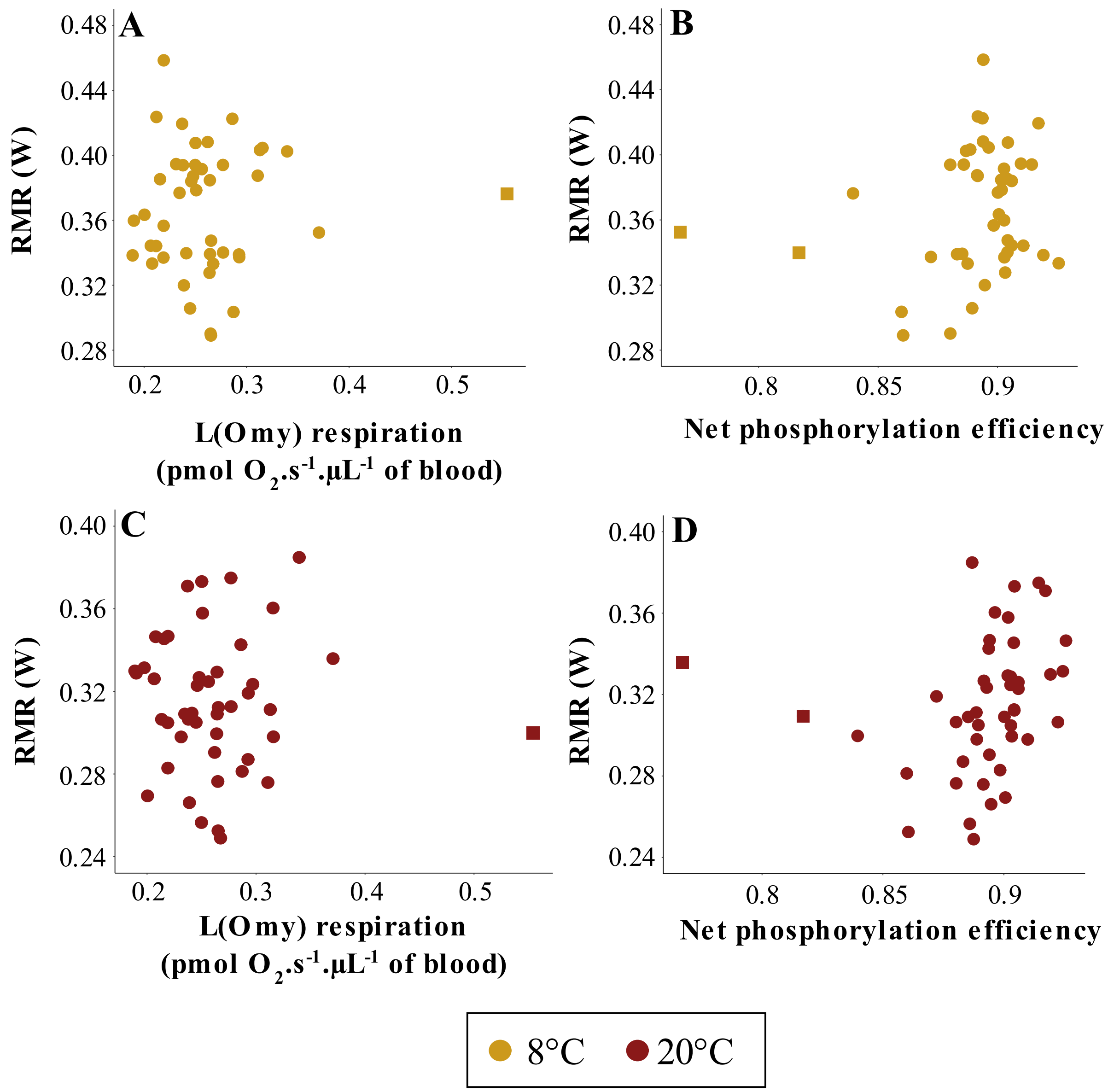

Fig. S5. Relationship between the resting metabolic rate (RMR) at 8°C or 20°C and mitochondrial respiration traits in permeabilized great tit blood cells when outliers (i.e., observations ≥ 2 standard deviations from the group mean, represented by squares) were retained in the analyses.

Biology Open • Supplementary information

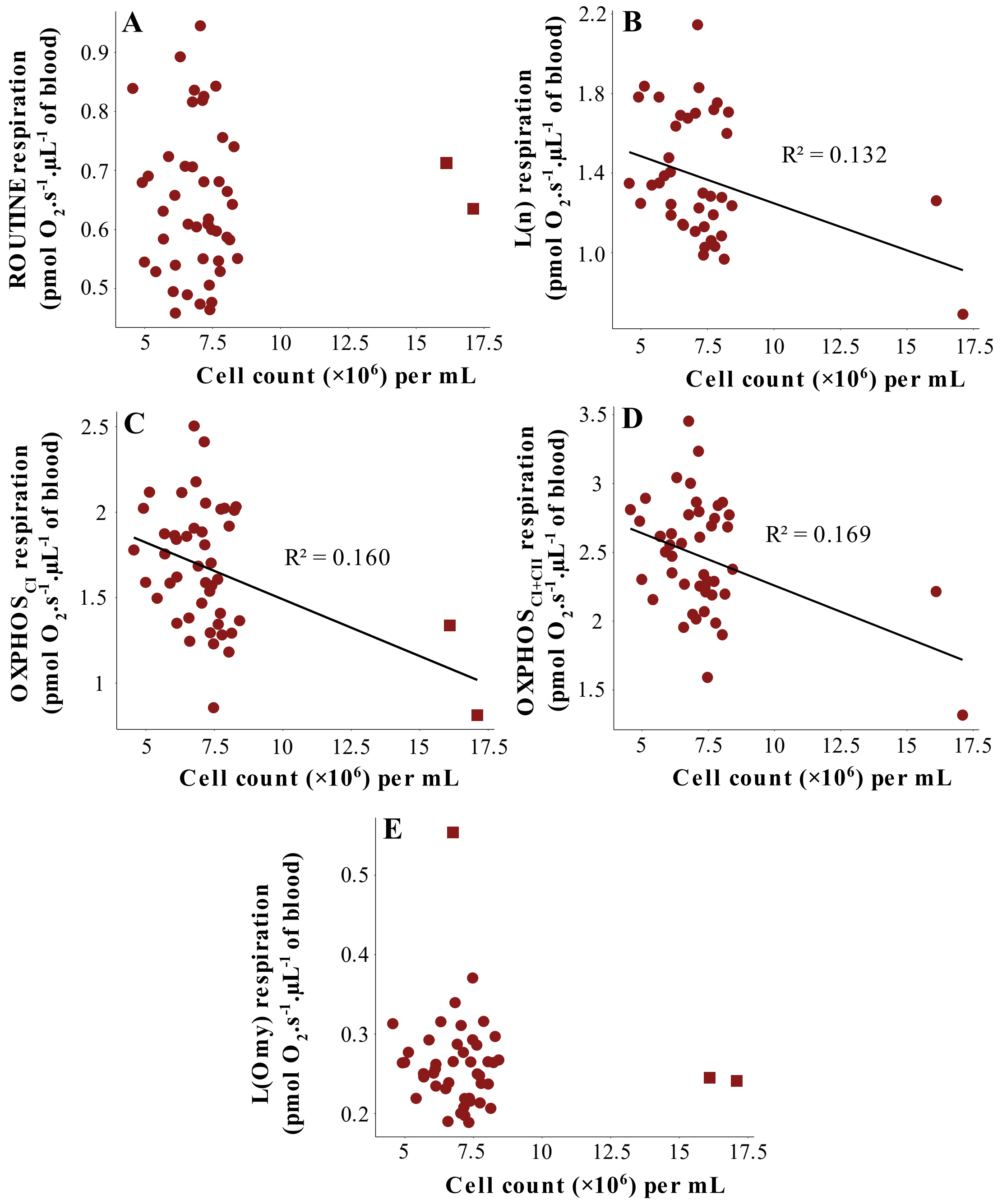

Fig. S6. Mitochondrial respiration rates as a function of cell count when outliers were kept in the models. Solid lines indicate significant relationships ($P \leq 0.05$). An observation was considered to be an outlier if it was more than 2 standard deviations from the group mean and are plotted as squares.

Biology Open • Supplementary information

Table S1. Parameter estimates from linear models of the relationship between resting metabolic rate (RMR) at 8°C and mitochondrial metabolism in permeabilized blood cells without outliers. Tabulated data correspond to the results shown in Figure S2. *P*-values in italics are ≤ 0.05. Six values are missing for the L(n) respiration since ADP was added directly after pyruvate and malate, preventing the measurement of L(n).

**Source of variation**	**Estimates ± s.e.m.**	**df**	***F*-value**	***P*-value**	***Multiple R^2***
**$RMR_{8°C}$**					
ROUTINE (n=44)	0.092 ± 0.049	1, 41	3.591	0.065	0.097
Body mass	-0.009 ± 0.006	1, 41	2.283	0.138	
L(n) (n=38)	0.027 ± 0.026	1, 35	1.1.071	0.308	0.033
Body mass	-0.006 ± 0.007	1, 35	0.723	0.401	
$OXPHOS_{CI}$ (n=44)	0.036 ± 0.017	1, 41	4.630	*0.037*	0.118
Body mass	-0.009 ± 0.006	1, 41	2.734	0.106	
$OXPHOS_{CI+CII}$ (n=44)	0.032 ± 0.014	1, 41	5.554	*0.023*	0.135
Body mass	-0.008 ± 0.005	1, 41	2.106	0.154	
L(Omy)* (n=43)	0.068 ± 0.159	1, 40	0.184	0.671	0.024
Body mass	-0.006 ± 0.006	1, 40	0.957	0.334	
Net phosphorylation efficiency* (n=42)	0.005 ± 0.004	1, 39	1.751	0.193	0.061
Body mass	-0.004 ± 0.006	1, 39	0.615	0.438	

Biology Open • Supplementary information

Table S2. Parameter estimates from linear models of the relationship between resting metabolic rate (RMR) at 8 or 20°C and mitochondrial metabolism in permeabilized blood cells, and for the relationship between mitochondrial respiration traits and cell count, when outliers were kept in the models. Tabulated data correspond to the results shown in Figures S3 and S4. *P*-values in italics are ≤ 0.05. Six values are missing for the L(n) respiration since ADP was added directly after pyruvate and malate, preventing the measurement of L(n).

**Source of variation**	**Estimates ± s.e.m.**	**df**	***F*-value**	***P*-value**	***Multiple R^2***
**$RMR_{8°C}$**					
L(Omy) (n=44)	0.058 ± 0.103	1, 41	0.318	0.576	0.026
Body mass	-0.006 ± 0.006	1, 41	0.973	0.330	
Net Phosphorylation Efficiency (n=44)	0.003 ± 0.002	1, 41	1.715	0.198	0.058
Body mass	-0.004 ± 0.005	1, 41	0.603	0.442	
**$RMR_{20°C}$**					
L(Omy) (n=46)	-0.016 ± 0.085	1, 43	0.037	0.849	0.015
Body mass	0.004 ± 0.005	1, 43	0.672	0.417	
Net phosphorylation efficiency (n=46)	0.003 ± 0.002	1, 43	2.048	0.160	0.059
Body mass	0.004 ± 0.005	1, 43	0.817	0.371	
**Cell count**					
ROUTINE (n=46)	0.001 ± 0.008	1, 44	0.012	0.912	0.000
L(n) (n=40)	-0.047 ± 0.020	1, 38	5.782	*0.021*	0.132
$OXPHOS_{CI}$ (n=46)	-0.066 ± 0.023	1, 44	8.401	*0.006*	0.160
$OXPHOS_{CI+CII}$ (n=46)	-0.076 ± 0.025	1, 44	8.914	*0.005*	0.169
L(Omy) (n=46)	-0.002 ± 0.004	1, 44	0.383	0.539	0.009

Biology Open • Supplementary information
